# Attitudes to possessions in emerging adults: Predictors of hoarding behaviours and beliefs

**DOI:** 10.1111/bjc.70003

**Published:** 2025-07-01

**Authors:** Enes Kartal, Jane Scott, Sharon Morein‐Zamir

**Affiliations:** ^1^ Possession and Hoarding Collective (POHOC), School of Psychology, Sport and Sensory Science Anglia Ruskin University Cambridge UK

**Keywords:** behaviour, belief, emerging adults, executive control, negative emotional response, hoarding

## Abstract

**Objective:**

Although hoarding symptoms are chronic and the average onset is late adolescence, younger cohorts have received little attention in research. Given the insidious symptom trajectory of hoarding and the unsatisfactory treatment outcomes in clinical groups, comprehensive research focusing on younger participants may reveal insights and suggest early intervention opportunities.

**Design:**

Cross‐sectional data were collected online from an emerging adult sample.

**Method:**

A total of 316 participants (aged 18–25) reported on hoarding symptoms, executive functioning, attention deficit/hyperactivity disorder (ADHD) symptoms, autism traits, obsessive‐compulsive disorder symptoms, social anxiety, psychological distress, emotion regulation, interpersonal attachment, and traumatic life events. Principal component analysis was used to cluster the data into underlying components.

**Results:**

Regression analysis showed that self‐reported executive control problems and negative emotional response are the key predictors of hoarding behaviours, with compulsivity and decisional impulsivity also being significant contributors. Importantly, the interaction between the two key predictors was not significant (*β* = .05, *p* = .273), implying independent contributions. Additionally, compulsivity, executive control and traumatic life events contributed to hoarding‐related beliefs.

**Conclusions:**

Difficulties in executive control, as noted in ADHD, would be an important target in the detection and intervention of hoarding symptoms among younger cohorts. Caution in the assessment of clutter in young people is needed as their control over common residential areas might be limited.

**Practitioner points**

- Self‐reported executive control problems and emotional difficulties were key contributors to hoarding behaviours. These two factors do not interact such that having problems in both areas did not amplify each other's impact.
- Targeted interventions depending on the individual's profile are recommended. Therapeutic strategies that enhance executive functioning, such as organizational training, problem‐solving exercises, and inhibition practice, may be helpful for individuals with predominant cognitive control difficulties. Individuals with emotional vulnerabilities may benefit more from attachment‐informed emotion regulation support.
- Trauma‐informed therapeutic approaches may help those who struggle with hoarding‐related beliefs.
- The assessment of clutter in young people should carefully consider their living conditions.

## INTRODUCTION

Holding on to possessions and having a cluttered household are common behaviours, but at extreme levels can hinder functioning and, when chronic and distressing, can constitute hoarding disorder (HD). Hoarding‐related symptoms include difficulty discarding, clutter, and excessive acquisition of items (Frost & Hartl, 1996). Hoarding symptoms are frequently comorbid with internalizing disorders such as major depressive disorder (MDD), anxiety disorders, obsessive‐compulsive disorder (OCD), and with neurodevelopmental conditions including attention deficit/hyperactivity disorder (ADHD) (Frost, Steketee, & Tolin, 2011; Lynch et al., 2015). Hoarding symptoms are present in 10% of adults in the general population and are associated with impairments in social functioning and cognition (Nutley et al., 2022). Research shows that hoarding follows a chronic course, with symptom severity increasing with time (Ayers et al., 2010). Treatment options are limited, with current treatment options yielding modest treatment gains (Tolin et al., 2025). Research on patients with HD has focused on older cohorts (Woody et al., 2014). However, hoarding begins on average during late adolescence (Zaboski et al., 2019) and younger age is related to better treatment outcomes (Tolin et al., 2015). Despite this, very little hoarding research has focused on younger populations. Investigating young cohorts would increase our understanding of hoarding and related risk factors at early stages. This could inform timely interventions to prevent the gradual increase in symptom severity and yield more effective outcomes, given pre‐treatment severity is currently the only clinical predictor reliably associated with better treatment response (Tolin et al., 2025).

Emerging adulthood in industrial societies encompasses young people aged 18–25 and is characterized by identity seeking, a sense of being “in‐between”, instability, self‐oriented attention, and perception of numerous possibilities (Tanner & Arnett, 2016). This period of life as a distinct phase is also important as it involves many changes and challenges, including key life events such as starting advanced education and training, finding a job, and transitioning away from the childhood home environment (Grob et al., 2001). Consequently, compared to earlier and later life stages, people are more likely to experience personality changes and the formation of habits related to independent living (Roberts et al., 2006). This age period is of key psychological importance also because it marks the common onset of many disorders, including anxiety disorders and OCD (Brakoulias et al., 2017; de Lijster et al., 2017). Adding to the inherent challenges of this period, there has been a significant and disproportionately greater increase in distress and mental health problems in emerging adults over the last decade, with the COVID‐19 pandemic and unprecedented social media use as potential contributors (Browning et al., 2022; Brunette et al., 2023; Duffy et al., 2019). Thus, research focusing on emerging adults can elucidate the development of hoarding symptoms in this critical life period.

Research and clinical evidence on HD and subclinical cohorts has linked hoarding with several behavioural, mental, and personality traits. As information processing deficits are one of the key factors in the cognitive behavioural model of the condition (Frost & Hartl, 1996), several studies have investigated these and, more broadly, executive functioning in young adults. Greater indecisiveness, concerns over memory, ADHD symptoms, impaired inhibition, and impulsivity have been reported by university students with high hoarding tendencies (Fitch & Cougle, 2013; Heffernan et al., 2023). Interestingly, some difficulties related to hoarding behaviours among emerging adults have not been replicated in older cohorts. For example, self‐reported impulsivity was not a unique predictor of hoarding behaviours in a large adult community sample (Morein‐Zamir et al., 2022), while it predicted hoarding behaviours among university students in the USA and Germany (Timpano et al., 2013).

More recently, research has highlighted additional psychological factors, such as adverse life events, personality traits, and emotional vulnerabilities in the development and maintenance of hoarding, resulting in new models, such as the biopsychosocial model (Tolin, 2023) and attachment model (Mathes et al., 2020). Anxious attachment has consistently been found to relate to hoarding behaviours among non‐clinical populations (Danet & Secouet, 2018; Kehoe & Egan, 2019; Neave et al., 2016). Research exploring the role of avoidant attachment appears mixed, although largely negative (Danet & Secouet, 2018; Keefer et al., 2012; Kehoe & Egan, 2019; Neave et al., 2016). A role for emotion regulation difficulties in hoarding is also evident among non‐clinical populations, with associations reported for anxiety sensitivity, distress intolerance, and negative urgency (Pardini et al., 2023; Phung et al., 2015). Contrary to impulsivity, the role of traumatic life events (TLEs) appears to increase from younger to older cohorts, with mixed results in university students (Shaw et al., 2016; Zhu & Geng, 2023) and significant results in community‐based studies (Mathes et al., 2018; Samuels et al., 2008) and older people with HD (Cromer et al., 2007; Landau et al., 2011). Social anxiety was also found to be related to hoarding behaviours among university students (Timpano et al., 2011), supporting the idea that the excessive object attachment seen in hoarding could be a compensatory response to insufficient functional interpersonal relationships (Yap & Grisham, 2021).

Erroneous beliefs about the nature of possessions have been proposed as central to the development and maintenance of hoarding (Frost & Hartl, 1996). These beliefs, as outlined in the cognitive behavioural model include: (I) intense emotional attachment and overreliance on possessions for emotional comfort and safety, (II) increased sense of responsibility towards items, (III) inflated need for control over possessions, and (IV) excessive concern about forgetting information if the related item is discarded (Steketee et al., 2003). It has been suggested that without addressing the nature and underlying factors contributiong to these erroneous beliefs, the development of effective treatments for hoarding is unlikely (Steketee & Frost, 2003). Indeed, a reduction in hoarding‐related beliefs has been linked to reduced symptom severity and impairment (Zakrzewski et al., 2022), highlighting their importance for interventions. Furthermore, these beliefs may contribute to the comorbid conditions frequently observed in individuals with hoarding symptoms, such as depression and anxiety (Steketee & Frost, 2003). Despite calls for further exploration of the nature and role of these beliefs, research examining them, particularly in younger cohorts, remains sparse. Examining the factors related to hoarding‐related beliefs, alongside behaviours in emerging adults may provide valuable insights into the developmental aspects of hoarding and help to identify early intervention points.

Although numerous psychological factors have been associated with hoarding behaviours among emerging adults, almost all are transdiagnostic and it remains unclear whether hoarding behaviours are predominantly driven by information processing and executive functioning difficulties or by emotional issues. Studies exploring the interactive effects of these two aspects would therefore be informative. Additionally, existing data on hoarding among emerging adults is derived almost exclusively from university students. It is difficult to extrapolate findings to the general population of emerging adults given that education level affects personality, general behaviours, and even hoarding‐specific behaviours such as saving (Dozier et al., 2016; Mammadov, 2022).

Therefore, the present study sought to investigate the relative contributions of various self‐reported psychological factors (executive functioning, emotional, behavioural, and experiential) to hoarding behaviours and beliefs among emerging adults. It also investigated whether the resulting factors related to executive function and emotional issues interact in this age group to predict more severe levels of hoarding‐related behaviour. We first subjected the data to principal component analyses (PCA) (Jolliffe & Cadima, 2016). We expected that components characterized by executive function difficulties and emotional issues would predict both hoarding behaviours and beliefs. We also anticipated that these components would interact super‐additively, amplifying each other's effect when both are present.

## METHOD

### Participants

Participants (*N* = 316) were recruited online from the general UK population (prolific.co.uk), with inclusion criteria being aged 18–25 and fluent in English. A quota sampling approach was employed to ensure gender balance. Recruitment commenced in January 2024, lasting 15 days.

The demographic characteristics of participants are presented in Table 1. The mean sample age was 22.71 (SD = 1.90) and most participants were White British. More than half were single and almost half reported living with other family members, with the average number of people living in the house being 3.42 (SD = 1.68). Participants reported diverse education levels, with around 32% not having university or equivalent degrees. The number of full‐time workers was high and only about a third were currently students.

**TABLE 1:** Demographic characteristics of participants.

Sample characteristics	*N*	%
Gender		
Women	153	48.4%
Men	153	48.4%
Non‐binary	10	3.2%
Ethnicity		
Asian	48	15.2%
Black	30	9.5%
Mixed	21	6.6%
White British	194	61.4%
White other or middle Eastern	20	6.3%
Other	3	.9%
Living arrangement		
Alone	22	7.0%
With partner	50	15.8%
With partner and with flatmates	3	.9%
With partner and with other family members	13	4.1%
With flatmates	72	22.8%
With other family members	152	48.1%
With other family members and flatmates	4	1.3%
Family status		
Married	10	3.2%
Partnered – living with partner	60	19.0%
Partnered – not living with partner	63	19.9%
Separated/Divorced	1	.3%
Single	182	57.6%
Education level		
GCSE	20	6.3%
A‐levels (12 years of school)	80	25.3%
Undergraduate degree/Apprenticeship or equivalent	161	50.9%
Postgraduate degree	55	17.4%
Employment status		
Looking after home/family	3	.9%
Student full‐time	72	22.8%
Student part‐time	6	1.9%
Part‐time work	41	13.0%
Working full‐time	133	42.1%
Part‐time work and student full‐time	13	4.1%
Part‐time work and student part‐time	1	.3%
Working full‐time and student full‐time	1	.3%
Unemployed	46	14.6%

### Procedure

Participants completed the survey online using the Gorilla Experiment Builder (Anwyl‐Irvine et al., 2020). Following informed consent, they answered the demographic questions followed by the questionnaires which were presented in a partially randomized order. Six attention check items were added throughout to detect inattentive responses (Meade & Craig, 2012). Participants received £5. The study had university ethics approval (ETH2324‐0603).

### Measures

#### Hoarding symptom severity

The Saving Inventory‐Revised (SI‐R) (Frost et al., 2004) comprises 23 questions and evaluates the three main aspects of hoarding behaviours: clutter, difficulty discarding, and acquisition. The scale showed excellent internal consistency, high test–retest reliability, and good discriminative and convergent validity (Frost et al., 2004). The SI‐R maintained its excellent internal consistency scores in the current study (*α* = .94), with subscales ranging from *α* = .85 to *α* = .91.

The Clutter Image Rating Inventory (CIR; Frost et al., 2008) further assessed clutter. The scale presents nine photographs of a number of rooms with gradually increasing clutter. Participants select the image most resembling their rooms. In this study, only images representing the bedroom were used, as control over other parts of the house might be limited for emerging adults. The CIR demonstrated good internal consistency, and good convergent and discriminant validity (Frost et al., 2008).

The Savings Cognitions Inventory (SCI) (Steketee et al., 2003) consists of 24 items pertaining to possession‐related thoughts. The SCI has four subscales: (I) emotional attachment; (II) memory; (III) control; and (IV) responsibility. The scale has excellent internal consistency, and good discriminative, convergent, and discriminant validity (Steketee et al., 2003), maintaining its internal consistency scores in the current study (*α* = .94) with subscales ranging from *α* = .67 to *α* = .93.

#### Executive functioning measures

The Behaviour Rating Inventory of Executive Function‐Adult Version (BRIEF‐A) (Roth et al., 2005) is a self‐report executive functioning measure. It consists of 75 items with nine subscales: (I) inhibit, (II) shift, (III) emotional control, (IV) self‐monitor, (V) initiate, (VI) working memory, (VII) plan/organize, (VIII) task monitor, and (IX) organization of materials. Moderate to high internal consistency scores for different subscales were found among the normative sample (Roth et al., 2005). The scale also has good convergent and discriminant validity (Roth et al., 2005; Wilson et al., 1997). In the current study, adequate to good internal consistency was found for the subscales (*α* values .78–.93).

Short Impulsive Behaviour Scale (UPPS) (Lynam, 2013) consists of 20 items tapping into five facets of impulsivity: negative urgency, lack of perseverance, lack of premeditation, sensation seeking, and positive urgency. The UPPS demonstrated adequate internal consistency and good convergent validity (Billieux et al., 2012; Cyders et al., 2014) with adequate to good internal consistency scores found for present data (*α* values .69–.83).

The Frost Indecisiveness Scale (FIS) (Frost & Shows, 1993) consists of 15 items and evaluates difficulties and attitudes during decision‐making. It has two subscales: fears about decision‐making and positive attitudes towards decision‐making. The scale has excellent internal consistency and good convergent validity (Frost & Shows, 1993; Rassin et al., 2007; Tuinstra et al., 2000). In the current study, high internal consistency scores were found for the subscales (*α* values .79–.81).

#### ADHD, autism, OCD, and social anxiety symptoms

The Adult ADHD Self‐Report Scale (ASRS) (Kessler et al., 2005) was utilized to assess ADHD symptom severity. The scale consists of 18 questions, with two subscales (inattention and hyperactivity/impulsivity). Continuous scoring was preferred to maintain statistical power (Cohen, 1988). The ASRS has high internal consistency, good test–retest reliability, and high convergent validity (Fuller‐Killgore et al., 2013; Gray et al., 2014). Present internal consistency was also high: inattention (*α* = .90) and hyperactivity/impulsivity (*α* = .86).

Autism Spectrum Quotient (AQ‐10) (Allison et al., 2012) measured autism symptom severity. The sensitivity score of AQ‐10 is .88, its specificity is .91, and it is strongly correlated with the long version AQ with 50 items (*r* = .92) (Allison et al., 2012). Furthermore, the AQ‐10 successfully discriminated people with autism spectrum disorder from control groups, with an area under the curve (AUC) of.9, suggesting high predictive validity (Booth et al., 2013). In the current study, the AQ‐10 had a Cronbach's alpha score of .75.

Obsessive Compulsive Inventory‐12 (OCI‐12; Abramovitch et al., 2021) was used to measure OCD symptom severity. The scale consists of 12 items and 4 subscales: checking, ordering, washing, and obsessing. The scale has good internal consistency, and convergent and discriminant validity (Abramovitch et al., 2021). In the current study, high internal consistency was found (*α* values .81–.87).

Social Interaction Anxiety Scale (SIAS) (Mattick & Clarke, 1998) measures fears and anxiety experienced before, during, and after social interactions. The scale has good internal consistency and discriminative and convergent validity (Mattick & Clarke, 1998; Osman et al., 1998). In the current study, excellent internal consistency was found (*α* = .95).

#### Psychological distress symptoms

To measure levels of psychological distress, the Depression, Anxiety, and Stress Scale‐21 (DASS‐21) was used (Lovibond & Lovibond, 1995). The scale has 21 items assessing anxiety, depression, and stress. The DASS‐21 has good to excellent internal consistency and construct validity across all three subscales (Antony et al., 1998; Clara et al., 2001). In the current study, high consistency scores were found for all subscales (*α* values .88–.93).

#### Emotional dysregulation symptoms

The Difficulties in Emotion Regulation Scale—Short Form (DERS‐SF) (Kaufman et al., 2016) measures emotion regulation. The scale contains 18 items and 6 subscales: (I) non‐acceptance; (II) goals; (III) impulse; (IV) awareness; (V) strategies; and (VI) clarity. The DERS‐SF has excellent internal consistency and good concurrent and discriminant validity (Burton et al., 2022; Kaufman et al., 2016; Victor & Klonsky, 2016). In the current study, high internal consistency scores were found (*α* values .80–.91).

#### Interpersonal attachment

The Adult Attachment Scale (AAS) was designed to assess adult attachment styles (Collins & Read, 1990). The scale consists of 18 items with three subscales: close, depend, and anxiety. The subscales of AAS have adequate internal reliability and good convergent and discriminant validity (Brophy et al., 2020; Collins & Read, 1990). Discriminant analysis showed that the dimensions of AAS clearly distinguish different attachment styles (Collins & Read, 1990). In the current study, Cronbach *α* values ranged from .71 to .86.

#### Traumatic life events (TLEs)

Life events checklist (LEC) screens for potential traumatic events (Weathers et al., 2013). It assesses exposure to 16 adverse life events such as natural disasters, combat or exposure to war, and sexual assault. In this study, standard scoring was used, as this method of scoring was found to be the most reliable (Weis et al., 2022). The scale has moderate test–retest reliability and good convergent validity (Gray et al., 2004; Kubany et al., 2000; Pugach et al., 2021). As each item of the scale screens different adverse life events, the internal consistency score was not calculated.

### Data analysis plan

Data was analysed using Jamovi version 2.3.26. Participants who failed more than three of the six attention checks were excluded. Descriptive statistics were computed for all variables of interest. Correlational analyses were conducted to examine the relationships between variables. PCA was performed on subscale scores for dimension reduction with Oblimin rotation as components were expected to correlate. To avoid over‐extracted components, parallel analysis (PA) was administered (Franklin et al., 1995). Barlett's test of sphericity and KMO measure of sampling adequacy were checked before components were interpreted.

Following component extraction, three primary multiple regression analyses were conducted to explore the predictors of hoarding behaviours and beliefs. The first model predicted SIR‐total, using all components derived from the PCA and, given its uniqueness, LEC scores as predictors. The second examined the interactive effect of the two PCA components representing executive and emotional factors on hoarding behaviours. The last model predicted SCI‐total with components and LEC scores as predictors. For all regression models, Cook's distance was inspected and variance inflation factor (VIF) values were checked for multicollinearity, with scores <2.1 deemed acceptable. In all regression models, predictor variables were mean centred. Bonferroni correction was applied for type‐I error control where multiple tests were carried out. The alpha level was adjusted by dividing the conventional threshold (*α* = .05) by the number of tests within each family of comparisons.

## RESULTS

### Data screening

Frequency tables and descriptive statistics were used for initial data inspection, with no missing data present throughout the dataset. Three participants failed more than three attention checks and were therefore excluded from further analysis. Outliers were identified via visual inspection (histograms, boxplots, and *Z*‐scores), and winsorizing was applied to seven variables that contained outliers with *Z*‐scores exceeding ±3. These included: SIR‐clutter, CIR‐bedroom, SCI‐emotional attachment, SCI‐responsibility, ASRS‐hyperactivity/impulsivity, SIAS total score, and LEC total score. Values below the 1st percentile and above the 99th percentile were replaced with the corresponding percentile value, adjusting <1.6% of the data for each variable. This method was chosen to reduce the influence of extreme scores without removing data, preserving the sample size while improving the robustness of parametric analyses (Wilcox, 2012).

### Descriptive statistics

Table 2 shows the descriptive statistics along with the Pearson correlation coefficients. Thirty‐eight participants (12%) scored above 43 on the SIR, which is the suggested clinical cut‐off for individuals under the age of 40 (Kellman‐McFarlane et al., 2019). Further, 7% of the sample scored above the cut‐off for clutter (≥17), 21% for difficulty discarding (≥15), and 31% above excessive acquisition (≥11). Generally, self‐reported executive functioning problems, impulsivity, ADHD symptom severity, and autistic traits had weak to moderate significant positive relationships with hoarding behaviours and beliefs. Indecisiveness had only a very weak to weak association with most hoarding measures, and no significant correlation with SIR‐clutter. OCD symptoms severity, social anxiety, psychological distress, emotion regulation problems, attachment anxiety, and exposure to TLEs had weak to moderate significant positive relationships with hoarding behaviours and beliefs, except CIR‐bedroom which did not correlate with OCD symptoms and TLEs. AAS‐depend and close subscales had very weak to weak negative correlations with hoarding symptoms. Among hoarding behaviours, clutter generally had lower associations with both self‐reported executive function difficulties and emotional factors than did difficulty discarding and acquisition.

**TABLE 2:** Descriptive statistics and intercorrelation (Pearson) matrix for variables of interest.

Variable	Mean	SD	Median	Range	1	1a	1b	1c	2	3	4	5	6	7	8	9	10	11	12	13	14	15	16
1. SIR‐Total	24.79	14.33	23.0	0–70	–																		
1a. Clutter	6.72	5.73	5.0	0–21	.85***	–																	
1b. Discarding	9.57	5.70	9.0	0–27	.88***	.59***	–																
1c. Acquisition	8.42	4.83	8.0	0–21	.87***	.60***	.72***	–															
2. CIR‐bedroom	1.78	.80	2.0	1–4	.47***	.48***	.38***	.32***	–														
3. SCI‐total	71.45	27.02	69.0	24–162	.62***	.42***	.68***	.54***	.24***	–													
4. BRIEF‐total	128.14	29.77	125.0	75–202	.55***	.36***	.55***	.55***	.41***	.44***	–												
5. UPPS‐total	43.85	7.48	44.0	23–65	.42***	.31***	.37***	.42***	.19***	.29***	.56***	–											
6. FIS‐total	44.78	10.44	44.5	18–72	.15**	.06	.21***	.15**	.15**	.19***	.34***	.21***	–										
7. ASRS‐total	31.49	13.35	31.0	2–71	.52***	.35***	.51***	.51***	.33***	.41***	.83***	.56***	.26***	–									
8. AQ‐total	4.10	2.34	4.0	0–10	.30***	.22***	.29***	.27***	.20***	.25***	.48***	.23***	.32***	.45***	–								
9. OCIR‐total	18.09	10.32	17.0	1–45	.43***	.30***	.43***	.38***	.11	.51***	.39***	.20***	.19***	.35***	.34***	–							
10. SIAS total	34.77	16.55	33.0	1–76	.40***	.20***	.45***	.39***	.21***	.37***	.60***	.18**	.33***	.49***	.50***	.40***	–						
11. DASS‐total	21.32	14.62	19.0	0–58	.48***	.32***	.48***	.45***	.29***	.41***	.70***	.39***	.32***	.58***	.39***	.54***	.60***	–					
12. DERS‐total	45.85	13.24	44.0	18–87	.48***	.34***	.45***	.47***	.35***.	.34***	.70***	.48***	.37***	.60***	.42***	.45***	.60***	.72***	–				
13.AAS‐anxiety	14.40	4.76	14.0	6–30	.46***	.37***	.40***	.43***	.23***	.34***	.47***	.40***	.27***	.35***	.26***	.28***	.40***	.46***	.52***	–			
14. AAS‐depend	17.10	5.15	17.0	6–30	−.23***	−.16**	−.20***	−.24***	−.18**	−.24***	−.36***	−.26***	−.10	−.33***	−.23***	−.29***	−.37***	−.47***	−.41***	−.36***	–		
15. AAS‐close	19.20	4.44	19.0	6–30	−.26***	−.19***	−.22***	−.29***	−.15**	−.23***	−.38***	−.22***	−.24***	−.38***	−.39***	−.27***	−.57***	−.42***	−.36***	−.33***	.52***	–	
16. LEC total	9.07	6.59	7.5	0–26	.28***	.20***	.28***	.27***	.11	.30***	.26***	.28***	.04	.23***	.000	.21***	.05	.28***	.25***	.24***	−.19***	−.09	–

*Note*: *N* = 316. For all questionnaires, higher scores imply great symptom severity and difficulties with the exception of AAS‐close and AAS‐depend, where higher scores denote secure interpersonal attachment style.

Abbreviations: AAS, The Adult Attachment Scale; AQ, Autism Spectrum Quotient; ASRS, Adult ADHD Self‐Report Scale; BRIEF, Behaviour Rating Inventory of Executive Function; CI*R*, Clutter Image Rating Inventory; DASS, Depression, Anxiety, and Stress Scale; DERS, Difficulties in Emotion Regulation Scale; Discarding, difficulty discarding; FIS, Frost Indecisiveness Scale; LEC, life events checklist; OCI*R*, Obsessive Compulsive Inventory‐Revised; SCI, Savings Cognitions Inventory; SIAS, Social Interaction Anxiety Scale; SI*R*, Saving Inventory‐Revised; UPPS, UPPS Impulsive Behaviour Scale—Short version.

***p < .001, **p < .01 (two‐tailed).

### Principal component analysis

The PCA yielded five components with eigenvalues >1, collectively accounting for 64.2% of the total variance. The first two components explained the largest proportion of variance, 23.73% and 18.68%, respectively. The remaining three components each accounted for between 6% and 9% of the variance. Table 3 provides the loadings for each component, the eigenvalues and the percentage of variance explained.

**TABLE 3:** Component loadings for the first five principle components.

Subscales	Components				
	Executive control	Negative emotional response	Compulsivity	Insecurity and self‐doubt	Decisional impulsivity
BRIEF‐working memory	.**95**	−.08	.07	.03	−.06
ASRS‐inattention	.**90**	−.02	−.01	.02	.01
BRIEF‐task monitor	.**90**	−.03	−.04	.04	.04
BRIEF‐plan/Organize	.**89**	.04	−.01	.04	.01
BRIEF‐initiate	.**79**	.16	−.06	.07	−.10
BRIEF‐inhibit	.**78**	.08	.01	−.07	.22
BRIEF‐organization of materials	.**77**	.11	−.19	−.04	−.01
ASRS‐hyperactivity/Impulsivity	.**66**	.08	.17	−.08	.20
BRIEF‐shift	.**62**	.28	.21	.02	−.20
BRIEF‐self‐monitor	.**60**	.14	.12	.01	.28
AQ‐total	.**42**	−.8	.30	.37	−.10
DERS‐strategies	.03	.**88**	−.05	.05	−.07
DERS‐non‐acceptance	−.13	.**84**	−.07	.02	.01
BRIEF‐emotional control	.21	.**70**	.04	−.11	−.02
DERS‐clarity	−.05	.**64**	.08	.24	.06
DERS‐impulse	.06	.**64**	.13	−.09	.32
DASS‐depression	.15	.**60**	.06	.20	−.08
DASS‐stress	.19	.**60**	.24	.03	−.03
DERS‐goals	.30	.**60**	−.11	−.06	−.16
DASS‐anxiety	.16	.**57**	.28	−.04	−.02
AAS‐anxiety	−.04	.**55**	−.06	.24	.21
UPPS‐negative urgency	.17	.**55**	.03	.07	.34
OCIR‐obsessing	.14	.**48**	.**43**	−.05	.01
OCIR‐ordering	−.01	.01	.**82**	.09	−.05
OCIR‐washing	.04	−.02	.**78**	.02	.01
OCIR‐checking	−.02	.02	.**74**	.01	.18
UPPS‐lack of perseverance	.34	−.13	**−.47**	.27	.17
FIS‐fears	.06	.07	.02	.**74**	−.06
FIS‐positive attitudes	.01	.15	.02	.**65**	−.23
DERS‐awareness	−.12	−.02	.01	.**65**	.36
AAS‐close	−.28	.02	−.21	**−.42**	.04
UPPS‐sensation seeking	−.02	−.15	.05	−.17	.**66**
UPPS‐positive urgency	.15	.21	.14	.06	.**65**
UPPS‐lack of premeditation	.34	.07	−.23	.17	.**54**
Eigenvalues	13.94	2.91	1.84	1.63	1.52
% of variance	23.73	18.68	8.93	6.74	6.15

*Note*: Factor loadings over .40 are bolded. Subscale scores were used in this analysis. For all scales, higher scores imply great symptom severity and difficulties except for AAS‐Close, where higher scores denote a higher likelihood of engaging in intimate relationships.

Abbreviations: AAS, Adult Attachment Scale; AQ, Autism Spectrum Quotient; ASRS, Adult ADHD Self‐Report Scale; BRIEF, Behaviour Rating Inventory of Executive Function; DASS, Depression, Anxiety, and Stress Scale; DERS, Difficulties in Emotion Regulation Scale; FIS, Frost Indecisiveness Scale; OCI*R*, Obsessive Compulsive Inventory‐Revised; UPPS, UPPS Impulsive Behaviour Scale ‐ Short version.

### Interpretation of principal components

*Executive control* loaded heavily on executive functioning difficulties, inattention, hyperactivity/impulsivity, and autism symptoms. *Negative emotional response* loaded on emotion regulation, psychological distress, anxious attachment, negative urgency, and obsessions. The third component, *Compulsivity*, loaded on compulsive OCD symptoms and lack of perseverance (negatively). *Insecurity and self‐doubt* loaded on indecisiveness, lack of emotional awareness and discomfort with intimate social relationships. The last component loaded on sensation seeking, positive urgency and lack of premeditation, reflecting the characteristics of *decisional impulsivity* (Dalley & Robbins, 2017).

The components showed low to moderate correlations, indicating that they represent related but distinct constructs. *Executive control* was correlated with *negative emotional response* (*r* = .599) and weakly correlated with *compulsivity* (*r* = .187), *insecurity and self‐doubt* (*r* = .283), and *decisional impulsivity* (*r* = .212). Similarly, *negative emotional response* also showed correlations with *compulsivity* (*r* = .336), *insecurity and self‐doubt* (*r* = .251), and *decisional impulsivity* (*r* = .123). *Compulsivity* was very weakly correlated with *insecurity and self‐doubt* (*r* = .060) and *decisional impulsivity* (*r* = .027). *Insecurity and self‐doubt* and *decisional impulsivity* were also very weakly correlated (*r* = .055).

AAS‐depend and LEC were not included in the final PCA model due to their high uniqueness scores, and SIAS due to its high loadings with multiple components (cross‐loading). The independent contribution of LEC to hoarding was examined by adding them to regression models along with the components. SIAS could not be retained due to its high multicollinearity.

### Predicting hoarding behaviours using principal components

The results of the multiple regression analysis predicting SIR‐total are displayed in Table 4. The results indicate that self‐reported executive control, negative emotional response, compulsivity, and decisional impulsivity were all significant predictors. Specifically, greater problems with executive control and higher negative emotional response were associated with increased hoarding‐related behaviours. Secondary analyses of SIR subscales (clutter, difficulty discarding, and acquisition) revealed similar associations, with the following exceptions. Compulsivity was not a significant predictor of clutter after applying type‐1 error correction (*β* = .11, *p* = .046). Decisional impulsivity did not predict difficulty discarding (*β* = .05, *p* = .308) and history of TLEs predicted difficulty discarding (*β* = .11, *p* = .019), but this effect did not survive type‐1 error control. Notably, the effect sizes of executive control difficulties (*χ* ^2^ (2) = .13, *p* = .94) and negative emotional response (*χ* ^2^ (2) = .01, *p* = .99) did not significantly differ between SIR subscales. An additional model predicting CIR‐bedroom indicated that only self‐reported executive control problems (*β* = .27, *p* < .001) and negative emotional response (*β* = .24, *p* < .001) were significant positive predictors, while compulsivity negatively predicted bedroom clutter (*β* = −.14, *p* = .010).

**TABLE 4:** Summary of regression analysis for SIR‐total with PCA components and LEC.

Predictor variables	Estimate	SE	95% CI		Beta	*p*
			LL	UL		
Intercept		1.13				**<.001**
Executive control	4.39	.82	2.78	5.60	.31	**<.001**
Negative emotional response	3.53	.85	1.86	5.19	.25	**<.001**
Compulsivity	2.38	.67	1.05	3.70	.17	**<.001**
Insecurity and self‐doubt	−.43	.67	−1.74	.89	−.03	.526
Decisional impulsivity	1.87	.66	.56	3.17	.13	.**005**
LEC	.19	.10	−.01	.40	.09	.064

*Note*: *F*(5, 315) = 33.6, *p* < .001; *R* ^2^_adj_ = .40. Bolded *p*‐values indicate statistical significance.

Abbreviations: Beta, standardized coefficients; CI, confidence interval; LEC, life events checklist; LL, lower limit; SE, standard error; UL, upper limit.

### Interaction between executive control and negative emotional response

Another analysis explored the standalone and interactive effects of the two key contributors, namely executive control and negative emotional response, to hoarding (*F* (3, 315) = 54.26, *p* < .001). The model accounted for approximately 34% of the variance in SIR‐total (*R* ^2^_adj_ = .34), again demonstrating significant effects for both executive control difficulties (*β* = .33, *p* < .001) and negative emotional response (*β* = .31, *p* < .001). However, the interaction term was not significant (*β* = .05, *p* = .273), indicating that the combined effect of executive control and negative emotional response did not add to hoarding behaviours beyond their individual contributions. Additional models predicting each SIR subscale (clutter, difficulty discarding and acquisition) indicated similar results.

### Predicting hoarding‐related beliefs using component scores

The results of the analysis predicting SCI‐total are provided in Table 5. Self‐reported compulsivity, executive control problems and exposure to TLEs were significant predictors of saving cognitions. Notably, negative emotional response and self‐reported decisional impulsivity did not significantly contribute to saving cognitions. Secondary analyses of SCI subscales (emotional attachment, memory, control and responsibility) revealed similar associations, except that history of TLEs did not predict memory (*β* = .11, *p* = .035) and control (*β* = .06, *p* = .253) after type‐1 error control was applied.

**TABLE 5:** Summary of regression analysis for SCI‐total with PCA components and LEC.

Predictor variables	Estimate	SE	95% CI		Beta	*p*
			LL	UL		
Intercept		2.21				**<.001**
Executive control	7.44	1.59	4.31	10.58	.28	**<.001**
Negative emotional response	1.02	1.65	−2.23	4.28	.04	.536
Compulsivity	9.80	1.32	7.21	12.39	.36	**<.001**
Insecurity and self‐doubt	.22	1.31	−2.35	2.79	.01	.864
Decisional impulsivity	1.61	1.29	−.93	4.15	.06	.214
LEC	.67	.20	.27	1.07	.16	.**001**

*Note*: *F*(5, 315) = 28.1, *p* < .001; *R* ^2^_adj_ = .35. Bolded *p*‐values indicate statistical significance.

Abbreviations: Beta, standardized coefficients; CI, confidence interval; LEC, life events checklist; LL, lower limit; SE, standard error; UL, upper limit.

## DISCUSSION

The current study indicates that self‐reported executive control problems and negative emotional factors contribute equally to hoarding behaviours among emerging adults. Contrary to our hypothesis, the two factors do not interact such that greater problems in both areas do not predict increased hoarding behaviours beyond their individual contributions. Moreover, we found self‐reported compulsivity, executive control problems, and exposure to TLEs to be significant predictors for saving cognitions or beliefs believed to be key to hoarding.

### Contributors to hoarding behaviours

Present findings point to a robust association between executive control and hoarding behaviours and traits. The executive control component encompassed self‐reported executive functions such as inhibition, organization and shifting among other functions, but also ADHD and autism symptoms. This broader construct is consistent with prior reporting of executive function difficulties being associated with hoarding behaviours in university students, including inattention, hyperactivity, impulsivity, cognitive inflexibility and disinhibition (Carbonella & Timpano, 2016; Fitch & Cougle, 2013; Heffernan et al., 2023; Timpano et al., 2013). It was predicted, given theorizing on information processing deficits (Steketee & Frost, 2003), that executive functioning difficulties might be related to clutter more strongly than difficulty discarding and acquisition. However, we found that executive control difficulties contribute similarly to all three hoarding subscales in emerging adults. The negative impact of clutter on functioning and well‐being tends to increase with age (Ferrari & Roster, 2018), suggesting the association between executive control and clutter might strengthen with age. Studies on the long‐term effects of personal clutter are needed, likely in longitudinal studies. There was a similar robust association between negative emotional response and all three aspects of hoarding behaviours and traits. This is consistent with studies reporting that psychological distress, attachment anxiety, and emotion regulation problems are positively associated with hoarding behaviours among non‐clinical populations (Morein‐Zamir et al., 2022; Neave et al., 2016; Timpano et al., 2014). Taken together, consistent with theoretical and empirical research on people with HD (Frost & Hartl, 1996; Kyrios et al., 2018), both self‐reported executive control and emotional problems contribute substantially to hoarding behaviours among emerging adults.

The current study identified two additional predictors of hoarding behaviours. Firstly, in keeping with diagnostic nosology, compulsivity—encompassing OCD symptoms such as ordering, washing, and checking, and perseverance—contributed to hoarding behaviours. This replicates studies on non‐clinical populations in adults (Morein‐Zamir et al., 2022; Taylor, 2017). Secondly, self‐reported decisional impulsivity, characterized by sensation seeking, positive urgency, and lack of premeditation, significantly contributed to hoarding behaviours. Consistent with this, self‐reported motor impulsivity, attentional impulsivity, urgency, and perseverance problems have been found as contributors among young adult samples (Fitch & Cougle, 2013; Timpano et al., 2013). On the other hand, studies exploring the broader construct of impulsivity among clinical samples have reported both significant (Hall et al., 2013; Hartl et al., 2005) and nonsignificant results (Sheppard et al., 2010; Tolin & Villavicencio, 2011). It appears that the role of impulsivity becomes less consistent in older and clinical cohorts, underlined by its developmental trajectory. Notably, decisional impulsivity predicted clutter and acquisition but not difficulty discarding in the present sample of emerging adults. In contemporary society, this cohort often faces significant pressure to purchase new items, driven by social media, peer influences, and targeted marketing strategies (Khan et al., 2016). This consumerist environment can exacerbate tendencies towards impulsive acquisition of items, particularly among individuals with higher decisional impulsivity. Notably, 31% of participants in the current study scored above the clinical threshold for acquisition on the hoarding measure. The excessive clutter observed in 7% of the current sample may reflect a long‐term consequence of excessive acquisition or difficulty discarding, each shaped by distinct psychological mechanisms.

Although decisional impulsivity predicted hoarding behaviours, the insecurity and self‐doubt component, comprising fears about decision‐making and positive attitudes towards decision‐making, did not have a unique contribution. While indecisiveness and self‐doubt have been considered key features of hoarding, particularly prior to the formal diagnostic separation of HD from OCD (Frost, Tolin, et al., 2011), more recent evidence from both clinical populations and undergraduate samples suggests that indecisiveness does not reliably predict hoarding behaviours, especially when other variables such as perfectionism are accounted for (Burgess et al., 2018; McCabe‐Bennett et al., 2025). A closer examination of the items comprising the decisional impulsivity and insecurity/self‐doubt components suggest that it may not be general self‐doubt or a negative orientation towards decision‐making that drives hoarding, but rather the impulsive decisions made under emotional arousal.

That both self‐reported executive control problems encompassing inattention and hyperactivity/impulsivity, and decisional impulsivity contribute to hoarding supports a link between ADHD and hoarding. Elevated hoarding behaviours have been reported among young and mid‐adulthood individuals with ADHD (Morein‐Zamir et al., 2022), and older people with HD reported higher ADHD symptoms in childhood than did people with OCD and controls (Worden & Tolin, 2023). ADHD is characterized by inattention and hyperactivity/impulsivity, with problems in organization, planning and working memory frequently seen among patients (Faraone et al., 2015). In general, hyperactivity/impulsivity symptoms tend to decrease with age, although inattention remains stable throughout the lifespan (Faraone et al., 2015). Consistent with symptom trajectory in ADHD, inattention is prominent in older people with HD (Tolin et al., 2018; Tolin & Villavicencio, 2011), while hyperactivity/impulsivity is inconsistently found. ADHD comorbidity has been highlighted in the hoarding literature in recent years, while the incidence of hoarding among people with ADHD has attracted less attention (Morein‐Zamir et al., 2022). Thus, research investigating hoarding among young people with ADHD would not only help to detect hoarding symptoms at an early age but also yield valuable information on maladaptive hoarding in this neurodevelopmental condition.

The effect sizes for executive control and negative emotional response were similar and substantial, highlighting the need to focus on these two main pathways. Notably, their interactive effect was nonsignificant. Increased difficulties in both are not associated with greater hoarding behaviours beyond each factor's individual contribution, contradicting the cognitive behavioural hoarding model (Frost & Hartl, 1996). Studies have confirmed a subgroup of HD characterized by unimpaired self‐reported executive control (Hall et al., 2013; Norberg et al., 2023), supporting an independent link with emotion. However, an independent executive functioning link in older people with HD is unlikely. Contributors to hoarding may change across the lifespan with emotional disturbance associated with long‐term executive control problems (Zainal & Newman, 2022) or hoarding symptoms (Ferrari & Roster, 2018) overshadowing cognitive control complaints as adults get older. This highlights further the need to understand their interplay across the lifespan.

Our findings also point to the complexity of using clutter as a measure of hoarding in emerging adults. Self‐reported executive control problems and negative emotional response predicted both SIR‐clutter and CIR‐bedroom, but self‐reported decisional impulsivity positively predicted only the former while compulsivity negatively predicted the latter. The two hoarding measures differ not only in stimuli (verbal vs. visual), and the extent of household areas involved but also the presence of distress associated with the clutter. With the majority of emerging adults living with others, they likely have limited control over common areas. The negative relationship between compulsivity and CIR‐bedroom is consistent with a compulsive ‘tidying’ dimension (Prudon, 2023). In parallel, compulsivity positively predicted SIR‐clutter, though this did not survive type‐1 error correction. It may be that emerging adults reporting greater repetitive cleaning, ordering and checking also experience greater distress over house‐wide clutter, though their bedrooms tend to be less cluttered. Therefore, the SIR might not be an accurate measure of clutter for emerging adults, highlighting the need to include the CIR or additional measures for those with limited control over their residential environment.

### Contributors to hoarding‐related beliefs

Erroneous beliefs about possessions are posited as central to hoarding behaviours and as predictors of poor treatment response (Steketee & Frost, 2003). Self‐reported compulsivity, executive control problems, and prior TLEs all uniquely contributed to hoarding‐related beliefs. The predictive role of compulsivity supports the idea that harm avoidance is central to both compulsive traits and hoarding beliefs (Gordon et al., 2013). Present results also align with previous findings that self‐reported inattention and distractibility contribute to hoarding‐related cognitions (Hallion et al., 2015; Tolin et al., 2018). The role of TLEs in cognitions is consistent with its role in emerging adults (Fontenelle et al., 2021) and older people with HD (Landau et al., 2011; Tolin et al., 2010). The relationship between TLEs and hoarding‐related beliefs established in young adulthood would be expected to influence evolving hoarding behaviours. Regarding emotional factors, our results diverge from those in older cohorts where anxiety and depressive symptoms, anxiety sensitivity, and negative urgency contributed to hoarding‐related beliefs (Chou et al., 2018; Phung et al., 2015; Zakrzewski et al., 2022). Hoarding is associated with substantial impairment and distress, leading to the depression and anxiety typically seen in older cohorts with HD (Frost et al., 2011). A better understanding of the causal relationships between different emotional and hoarding components would yield more effective intervention options.

### Future directions, strengths and limitations

The existence of two distinct contributors to hoarding among emerging adults suggests the possible utility of independent specific intervention strategies for either, particularly in younger cohorts. Targeted interventions depending on the patient profile may improve treatment outcomes and dropout rates seen in hoarding treatment (Williams & Viscusi, 2016). Those with primarily cognitive control difficulties may benefit from treatment strategies supporting their executive functioning, including organizational training, problem‐solving, and inhibition practice, while individuals with emotional vulnerabilities may benefit from attachment‐informed emotion regulation support. Additionally, trauma‐informed therapy may particularly help those who struggle with hoarding beliefs encompassing emotional attachment and responsibility. Given the low treatment response rate of older people with HD, personalized early intervention strategies are urgently needed.

To our knowledge, this is the first study exploring the interactive effects of self‐reported executive control and emotional factors in hoarding spanning a large array of established measures in a well‐powered dataset in emerging adults encompassing non‐students. By including non‐university students, the sample may better reflect the diversity of the general population of emerging adults, potentially capturing factors associated with hoarding that might remain unexplored in student‐only samples. However, the cross‐sectional study design prevents conclusions regarding temporal and causal relationships. This study also investigated a UK‐based specific age group (18–25), suggesting caution when generalizing the results to other populations. Emerging evidence suggests that cultural and economic factors might influence hoarding‐related behaviours, including acquisition and discarding (Cui et al., 2024; Tolin, 2023). In the context of emerging adulthood, factors such as financial instability, prolonged dependence on family, and identity exploration may also differ across cultures, possibly affecting the presentation of hoarding tendencies in this cohort (Nelson et al., 2004; Su et al., 2022). While we assessed social anxiety symptoms, due to its cross‐loadings and multicollinearity we could not inspect this construct in more detail. Further, the present study relied exclusively on self‐report. Although self‐reported dysfunction is an important predictor of hoarding symptom severity (Zakrzewski et al., 2022), multi‐method approaches including behavioural and independent raters would further contribute to our understanding of hoarding symptoms among emerging adults.

## CONCLUSION

In summary, this study focused on a critical period of life stage in terms of the development of hoarding symptoms. The results suggest that executive control problems and negative emotional response are specifically important in hoarding behaviours. Notably, the interaction between these two factors is not significant, implying two distinct main contributors associated with hoarding behaviours among emerging adults. In addition, we found that self‐reported compulsivity, executive control problems and exposure to TLEs contribute to hoarding‐related beliefs. Further research with multi‐method approaches would increase our understanding of the hoarding phenomenon in this critical age group.

## AUTHOR CONTRIBUTIONS

**Enes Kartal:** Conceptualization; investigation; methodology; writing – original draft; writing – review and editing; data curation; formal analysis. **Jane Scott:** Conceptualization; investigation; writing – review and editing; supervision; methodology. **Sharon Morein‐Zamir:** Conceptualization; investigation; methodology; writing – review and editing; supervision; formal analysis.

## CONFLICT OF INTEREST STATEMENT

The authors declare no conflict of interest.

## Data Availability

Data will be made available on request.
